# Neuronal Avalanches Differ from Wakefulness to Deep Sleep – Evidence from Intracranial Depth Recordings in Humans

**DOI:** 10.1371/journal.pcbi.1002985

**Published:** 2013-03-21

**Authors:** Viola Priesemann, Mario Valderrama, Michael Wibral, Michel Le Van Quyen

**Affiliations:** 1 Max Planck Institute for Brain Research, Department of Neural Systems and Coding, Frankfurt, Germany; 2 University of Los Andes, Bogotá, Colombia; 3 Johann Wolfgang Goethe University, Magnetoencephalography Unit, Brain Imaging Center, Frankfurt am Main, Germany; 4 Hôpital de la Pitié-Salpêtrière, Centre de Recherche de l'Institut du Cerveau et de la Moelle épinière (CRICM), INSERM UMRS 975 - CNRS UMR 7225-UPMC, Paris, France; Indiana University, United States of America

## Abstract

Neuronal activity differs between wakefulness and sleep states. In contrast, an attractor state, called self-organized critical (SOC), was proposed to govern brain dynamics because it allows for optimal information coding. But is the human brain SOC for each vigilance state despite the variations in neuronal dynamics? We characterized neuronal avalanches – spatiotemporal waves of enhanced activity - from dense intracranial depth recordings in humans. We showed that avalanche distributions closely follow a power law – the hallmark feature of SOC - for each vigilance state. However, avalanches clearly differ with vigilance states: slow wave sleep (SWS) shows large avalanches, wakefulness intermediate, and rapid eye movement (REM) sleep small ones. Our SOC model, together with the data, suggested first that the differences are mediated by global but tiny changes in synaptic strength, and second, that the changes with vigilance states reflect small deviations from criticality to the subcritical regime, implying that the human brain does not operate at criticality proper but close to SOC. Independent of criticality, the analysis confirms that SWS shows increased correlations between cortical areas, and reveals that REM sleep shows more fragmented cortical dynamics.

## Author Summary

Brain activity shows complex dynamics, even in the absence of external stimulation. In fact, most brain activity is generated internally. Therefore, it is crucial to understand the generation principles of internal activity. One hypothesis is that complex brain dynamics emerges from simple local interactions if the network is in a specific state, called “self-organized critical” (SOC). SOC indeed can account for dynamics in slices of brain tissue. However, we lack evidence that human brain dynamics is SOC. In addition, we wondered whether SOC can account for brain activity from wakefulness to deep sleep, despite clear changes in brain dynamics with vigilances states. To answer these questions, we analyzed intracranial depth recordings in humans. We found evidence that the human brain indeed operates close to criticality from wakefulness to deep sleep. However, we found deviations from criticality with vigilance states. These deviations, together with our modelling results, indicated that the human brain is close to SOC, but in a subcritical regime. In the subcritical regime complex dynamics still emerges from purely local interactions, but are more stable than the SOC state. In fact, operation the subcritical regime allows for a safety margin to supercriticality, which was linked to epilepsy.

## Introduction

Distinct patterns of neuronal dynamics are observed across vigilance states as the brain transitions from wakefulness to sleep [1]. In contrast, a specific attractor state, called self-organized critical (SOC), has been proposed to govern brain dynamics, because models suggest that the SOC state allows the brain to operate both flexibly and reliably, and allows for optimal information coding, processing and storage [2]–[4]. But does the brain always operate in the SOC state, despite wide variations in the neuronal dynamics across vigilance states, or does the brain – in the framework of critical dynamics – undergo a state transition away from the critical to subcritical or supercritical states [5]–[9]? The critical state may be optimal for information processing and storage; however, during sleep the brain might not be in a state of optimal processing capacities, since sleep dynamics might equally be optimized to save energy, to restore tissue, for synaptic homeostasis, for thermoregulation, or for plasticity, learning and memory [10]–[14]. Thus there are many reasons why the brain might not be in a critical state during sleep.

An observation of deviations from the critical state for certain vigilance states would also imply phase transitions between vigilance states in the context of SOC. Evidence for phase transitions has been found *in vitro* and *in silicio* [5]–[9], [15]–[17]. These findings demonstrate that a neural network is in principle capable to undergo such transitions. However, we have no evidence yet for phase transitions to sub- or supercriticality *in vivo*.

An investigation of such phase transitions, and the critical state proper, requires sufficient temporal and spatial sampling, since SOC dynamics involves the entire system and not just a subset. As a consequence, power law relationships – the hall mark feature of SOC - are only reliably recovered under sufficient sampling [18]. Thus, classifying sub-, supercritical and critical states in heavily subsampled system becomes difficult. Therefore, we here used local field potentials (LFP) recorded with intracranial depth electrodes from epileptic patients. These recordings sampled activity with up to 61 contacts distributed across the entire brain. In contrast to conventional electroencephalography (EEG) or electrocorticography (ECoG) which sample from the surface of the head or brain, the LFP electrodes extended into deep brain structures and sample the activity locally from the surrounding tissue. These recordings allowed us to sample not only superficially (as with EEG) or locally from a single brain area (as with implanted electrode arrays), but to record activity from many brain areas in parallel.

**Figure 1:**
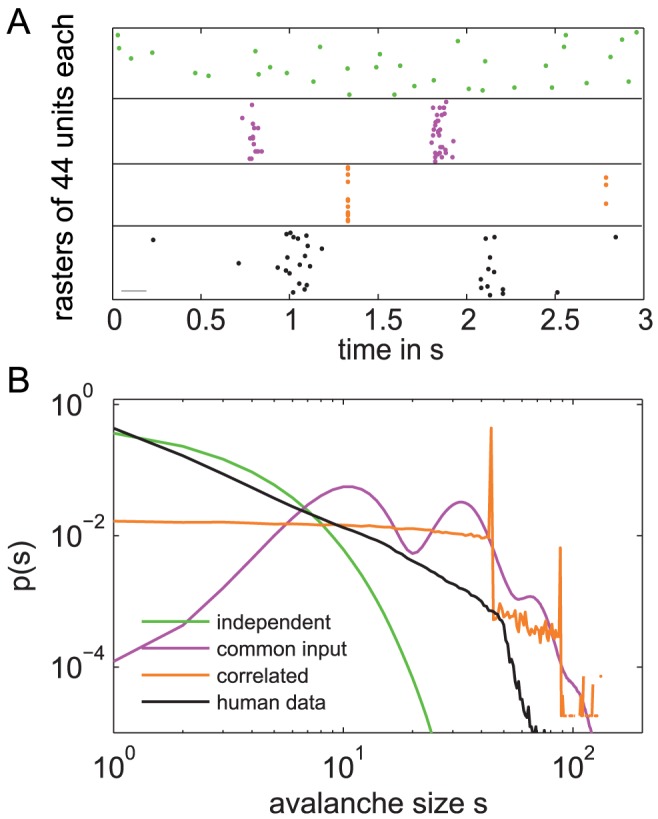
The global correlation structure between units is reflected in the avalanche distribution. **A.** Each of the four raster plots depicts events from a different stochastic process with 44 units each. Each process had the same event rate (¼Hz) but different correlations structures between its 44 units: independent Poisson processes (green), stochastic input to two different subsets of the units (pink), and high correlation between units (orange). The black dots represent events recorded from the human brain (44 electrodes, ¼Hz event rate). The horizontal gray line depicts the bin size applied to get the *p(s)* in (B). **B.** Each of the avalanche size distributions *p(s)* corresponds to one of the processes in (A). *p(s)* reflects the correlation structure of the data. High correlations resulted in more large avalanches (orange, pink), while the Poisson processes show *f(s)* close to an exponential (green). However, here only *p(s)* from the human data (black) showed a power law.

To estimate whether the neuronal dynamics in the human brain operated close to criticality, or in sub- or supercritical states, one has to extract spatio-temporal clusters of enhanced activity, called *neuronal avalanches* [19]. The size distribution of these neuronal avalanches reflects the spatio-temporal correlation structure between recording sites. This correlation structure can be organized in various ways (Figure 1A): Units can be independent or highly correlated. The correlation structure can form specific clusters (e.g. showing high correlations between brain areas of the same modality), or it can be anywhere between these extremes. For each of these classes, the event distributions over time (Figure 1A) and the resulting avalanche size distributions *f(s)* look very different (Figure 1B). Notably, only for very specific correlation structures, the avalanche distributions show a power law (black). In this case, *f(s)* shows more large avalanches than a system of uncorrelated units, however, it does not prefer any specific avalanche size. Therefore, power law distributions are termed scale free. A power law indicates that the activity between the units is correlated, but the units don't form strongly interconnected subgroups. Thus, only under very specific conditions, the avalanche distributions follow a power law, which is then indicative for the SOC state.

To assess SOC across vigilance states in humans, we evaluated neuronal avalanches from five patients, two nights each and for each vigilance state separately. We found that neuronal avalanches across brain areas indeed were best described by a power law, indicative of the SOC state. This even held for each of the vigilance states separately, although each state is characterized by distinct neuronal dynamics. However, the avalanche distributions differed slightly but consistently between vigilance states. Slow wave sleep (SWS) showed the largest avalanches, wakefulness showed intermediate ones, and rapid eye movement sleep (REM) showed the smallest. These differences in avalanche distributions implied that not all vigilance states can be SOC. In fact, the data together with modeling results indicated that the human brain operates close to criticality but within the subcritical regime. Within the subcritical regime, the differences between vigilance states may be mediated by tiny changes in effective synaptic strength. These changes tune the brain closer to criticality (SWS) or farther away (REM). – Independent of the framework of criticality, the avalanche measures confirmed that SWS shows increased correlations between cortical areas [20], and they revealed a new phenomenon, namely that REM sleep is characterized by more fragmented cortical dynamics than SWS and wakefulness.

## Results

### Neuronal avalanche distributions across the human brain are close to a power law

**Figure 2:**
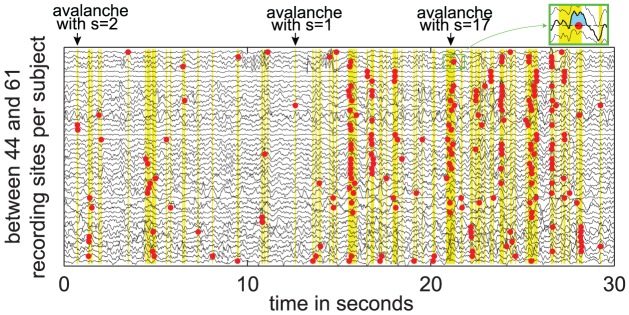
Definition of neuronal avalanches. Black traces show LFP from 44 parallel intracranial depth recording sites in one patient. For each recording site the area under the deflection lobe between two zero crossings was calculated (see green box – blue indicates the area under the deflection lobe). A binary event (red dot) was counted by selecting the biggest area values such, that each recording site during each phase of constant sleep stage had an event rate of exactly ¼ Hz (in this example). The binary events across recording sites occurred in clusters (yellow background). These clusters are called neuronal avalanches. The avalanches were separated by pauses of no activity (white background). The avalanche size *s* is defined as the total number of binary events in one cluster. As examples, the sizes *s* of three avalanches were indicated above the raw traces.

Neuronal avalanches are spatio- temporal clusters of enhanced activity that can span the entire system but can also be restricted to a single site only (Figure 2). The size *s* of a neuronal avalanche is defined as the total number of recording sites that show enhanced activity during one avalanche [19]. We sampled LFP with up to 61 intracranial depth recording sites (Figure 2). The intracranial depth electrodes are shaft electrodes with several spatially separated contacts per shaft. Each shaft was placed separately in the brain and targeted areas such as the hippocampus and the amygdala, depending on the specific clinical needs. For the LFP, which is supposed to reflect mainly synaptic currents [21], [22], we extracted neuronal avalanches on the base of the size of the LFP deflection lobes (see Methods, see Figure 2) and estimated their sizes *s*, their duration *d*, and their characteristic branching parameter σ as described in [19].

**Figure 3:**
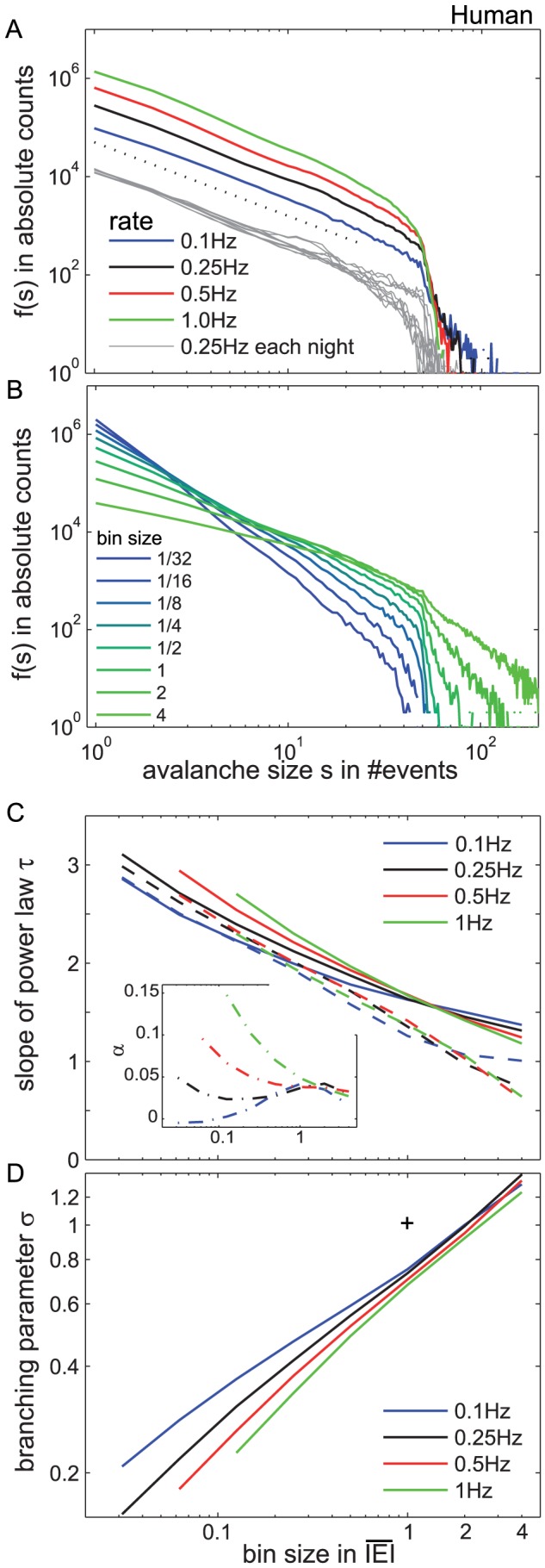
The neuronal avalanche size distribution *f(s)* for humans approximated a power law. **A.** The colored line shows *f(s)* for all avalanches across all 10 nights, evaluated at different event rates and at *bs = 1*·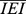. The gray lines show *f(s)* at *r* = ¼Hz separately for each of the nights to indicate the variability between recording nights and patients. For better visibility, the gray distributions have some offset, while the colored distributions all are in absolute counts. *f(s)* approximated a power law (τ = *1.5* was indicated by the dotted line). The cut off around *s* = *50* is known to coincide with the number of recording electrodes, 51 on average. **B.** The slope of *f(s)* changed with the temporal scale or bin sizes (*bs*). The *bs* was between *1/32* 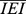 and *4* 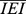, while here *r* was fixed at *r* = ¼ Hz. With larger *bs*, the slope of *f(s)* became flatter, but the distributions always resembled a power law. **C.** The slope τ of *f(s)* depended on the bin size (*bs*), but little on the rate (colored lines). The full lines show τ from fitting a power law, while the dashed lines show τ and α for a power law with cutoff (see inset for α). τ and α for small *bs* at high rates are not defined, because the *bs* there became smaller than the time resolution from sampling (*2.5 ms*). Estimation errors for τ and α scale with *n* ^−½^ where *n* is the number of samples [24]. Here, *n*≈*10^6^*, and thus the error is of the order *10^−3^*, and thus error bars are close to line thickness. For details on the fitting parameters and quality, see also Supplementary Table S1 and Figure S1. **D.** The branching parameter *σ* was plotted over the *bs*. *σ* changed with the *bs*, but was similar across event rates (colored lines). The (+) depicts [*σ = 1, bs = 1*] for visual guidance.

Figure 3A shows the avalanche size distributions *f(s)* for all patients, recording nights and vigilance states combined. *f(s)* consistently resembled a power law independent of the underlying event rate (Figure 3A). Note that imposing an event rate is basically equivalent to the common application of a threshold, however, it allows for more precise control over the contribution of each site to the avalanches. The avalanche distributions extended until *s*≈*50* and showed a drop for larger *s*. Based on previous work, we expect the drop to occur at around the total number of sampling sites, which here is at *N*≈*50* [18], [19], [23]. The function, which accounted best for the distributions with the drop, was a power law with cutoff (supplementary material S1):
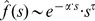
(1)We applied maximum likelihood estimation [24], to estimate the parameters τ and α. τ = *1.41±0.06* (mean ± std) and *α = 0.028±0.009*. When fitting a power law proper, we obtained τ = *1.58±0.06*. The slope τ of *f(s)* did hardly change despite a tenfold change in the event rate (Figure 3C). This apparent invariance of τ against a change in event rate shows that the threshold proper does not influence the avalanche dynamics, or stating it differently, there are no characteristic deflection lobe sizes which introduce their own dynamics or pattern sizes to the avalanche distribution.

The above results were evaluated at one specific temporal scale or bin size (*bs*), namely at a bin size of one average inter event interval (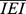), i.e *bs = 1*•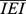. A change of the temporal scale – in contrast to the rate – had a clear influence on the slope of *f(s)* (Figure 3B, evaluated for *r = ¼ Hz*). This is a trivial effect, since larger *bs* allowed to combine more events to a single avalanche, thereby reducing the number of small and increasing the number of large avalanches. Nonetheless, it was surprising to find that the power law behaviour was not destroyed over the more than *100* fold change of time scales (Figure 3B). Fitting a power law to *f(s)* showed a clear decrease of the slope from τ≈*3.1* to τ≈*1.3* with the *bs* (Figure 3C). The dependence of τ on the *bs* was similar across all rates for both model functions - fitting a power law proper and a power law with cutoff. This indicated again that the temporal scale has a major influence on the avalanche distribution, while the threshold has little impact. A power law distribution for the avalanche sizes across a wide range of parameters (*bs* and *r*) suggests that cortical dynamics across brain areas are close to the critical state.

Another parameter which characterizes neuronal avalanches is the branching parameter σ. The branching parameter is a measure to quantify whether a process expands (σ>*1*) or diminishes (σ<*1*). More precisely, σ is defined as the expected number of events which are triggered by a single event [15]. We found that σ clearly changed with the temporal scale (*bs*), while it changed only little with the rate (Figure 3D). This is in line with previous studies [19]. However, while these studies reported σ to be close to one for *bs = 1*·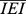, we here found σ to be a little smaller than one at *bs = 1*·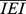, hinting at a slightly subcritical state of operation.

As mentioned above, the electrodes were placed individually in each patient. Most contacts were placed in the neocortex (NC) while a few contacts in each patient recorded from the amygdala and the hippocampus (AH). To understand whether NC contacts contributed differently to the avalanches compared to AH contacts, we tested whether the contribution of a single contact to avalanches of size *s* depended on the electrode location. Or saying it differently, for the events of each contact we estimated the probability to participate in avalanches of size *s*. The contributions to avalanches of size *s* did not differ between NC and AH contacts (cluster based randomization test, *p = 0.30* T-metric [25]). Thus the AH contacts contributed in the same way to avalanches of each size as the NC contacts. This, to our knowledge, is the first report that non- neocortical brain areas also contribute to neuronal avalanches.

### Comparison of the experimental results with a simple SOC model

To better understand our results concerning approximate power law distributions in the brain, we simulated model avalanches to study effects of finite size and subsampling. We chose an integrate- and- fire SOC model (SOCM) [26] that shows recurrent activity, runs in a 3D volume, has a refractory period, and is capable of reproducing neuronal avalanches recorded in monkeys [18]. In this model the activity propagates via next neighbor connections: A unit “integrates” all “energy” it receives from its next neighbors until the energy level crosses a certain threshold. It then releases the energy to its next neighbors without loss (it “fires”). The total number of subsequent “firing” events is defined as the avalanche size (see Methods). Although this SOCM is very simple, it bears similarity to the brain: first, its threshold action resembles the “integrate and fire” mechanism that characterizes neural signal generation, and second, its local connectivity resembles the dominance of local connections in the brain [27], [28]. Most importantly, this particular model allowed us to control the signal propagation efficacy (representing effective synaptic strength), which tunes the model to sub- and supercritical states, for comparison to the data.

**Figure 4:**
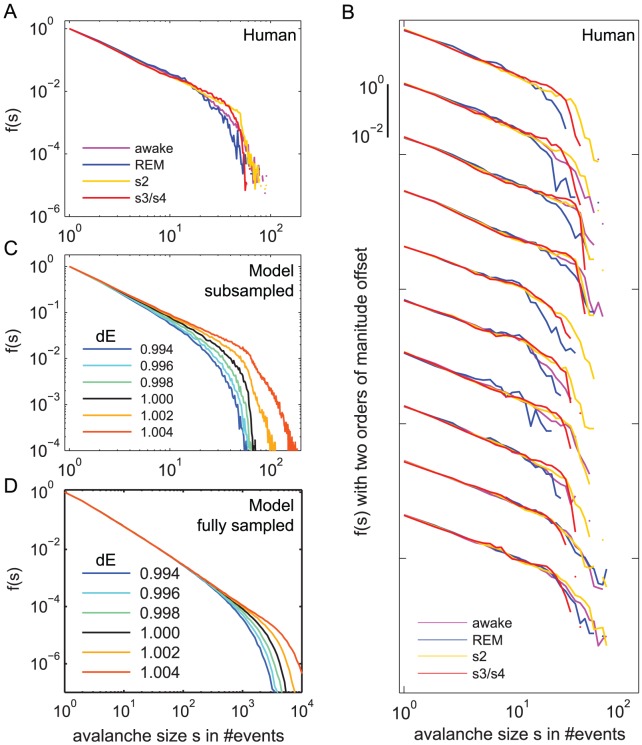
Avalanche distributions differed with vigilance states and synaptic strength *dE*. **A.** The avalanche size distribution *f(s)* for the neuronal avalanches was evaluated for each vigilance state separately. *f(s)* was similar for all vigilance states, however, it showed fewer large avalanches for REM sleep than for SWS (s2 and s3/s4). All *f(s)* were normalized such that *f(s = 1): = 1*. **B** Here, we showed the same results as in A, however, *f(s)* was plotted separately for each of the 10 recording nights to show that the differences in *f(s)* with vigilance states were present in each night. (Logarithmic binning to smooth the curves; the offset between the sets is two orders of magnitude.) **C.** The avalanche distribution for the subsampled SOC model was close to a power law for the critical state (black line). To deviate from the critical state, the synaptic strength *dE* was varied systematically by up to *0.6%* (colored lines). Technically, for *dE*<1 the model is subcritcal and for *dE*>1 it is supercritical. With larger *dE*, *f(s)* showed an increased number of large avalanches. For the supercritical state (*dE*>1) a significant amount of avalanches was larger than 64, the number of sampling sites. **D.** The results for the fully sampled model look similar to the subsampled model, except that the cutoff is, as expected, at larger *s*.

The SOCM in its critical state produced avalanche distributions that closely resembled those observed in the data, which is, *f(s)* approximated a power law for avalanche sizes up to the number of sampled sites (*N = 25^3^ = 15625*), then showed a drop off (Figure 4D black trace). This drop off is due to the finite size of the model [26]. To better compare the modeling results to our experimental data, where up to 61 sites were sampled, we adjusted the number of recording sites in the model, i.e. we took into account only the activity of a small subset of the model sites (*4×4×4 = 64*) and dismissed the activity of all the other sites (‘subsampling’). In this case, *f(s)* from the subsampled model had a drop off at around *s* = *64*, the total number of sampled sites (Figure 4C, black trace). By analogy, we expect the drop off of the neuronal *f(s)* to be caused by the limited number of recording sites [18]. With even more recording sites, we expect the distribution of the neuronal avalanches to extend over more orders of magnitude.

SOC models showed power laws with cutoff for their avalanche distributions [26], [29], [30]. This is well known, however, we still wanted to test this statistically [24]. Indeed, we confirmed that a power law with cutoff provided a better fit than alternative functions for both, the fully sampled and the subsampled model *f(s)* (supplementary table S1). However, though the power law with cutoff provided the best fit, it was not sufficient to pass a statistical test proposed by Clauset and colleagues. The same results were obtained for *f(s)* from the human brain (supplementary table S1). Thus, neither the neuronal avalanches from the human brain, nor the avalanches from the SOC model followed a power law with cutoff in the strict sense.

**Figure 5:**
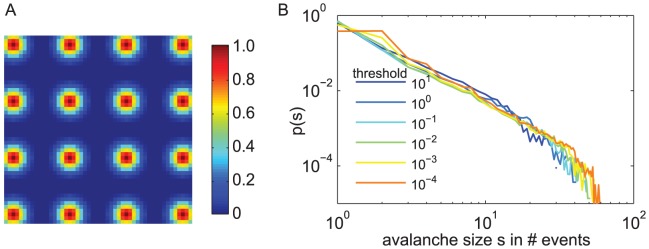
Avalanches from virtual LFP signals of the “spiking” SOC model showed a power law. **A.** We sampled virtual LFPs from the SOC model with 4×4×4 = 64 virtual electrodes. Each virtual electrode sampled from a 3D sphere centred on the electrode tip. The sampling weights for a slice with 4×4 electrodes are indicated here in colour. **B.** The avalanche size distributions *p(s)* on the 64 virtual electrodes showed a power law for a wide range of thresholds (coloured traces). For the virtual LFP, avalanches were calculated the same way as for the real LFP: Whenever the area under a deflection lobe exceeded a certain threshold, a binary event was attributed. *p(s)* was more noisy for higher thresholds since less events contributed to the distribution.

We here compared neuronal avalanches from LFP recordings in humans to avalanches of a spiking neuronal model, as this latter model is known to be SOC [26]. We may still ask, whether both scales may reflect the same phenomenon. To answer this question we sampled activity in our spiking model with virtual LFP electrodes. Each electrode sampled activity from multiple sites (Figure 5A). We then analyzed the virtual LFP in the same way as the human LFP. Indeed, just like the spiking model, the virtual LFP activity showed a power law for its avalanche dynamics, although with a steeper slope (Figure 5B). Hence, the simplest explanation is that scale-free LFP dynamics reflect the underlying SOC dynamics of spike avalanches.

### Scaling laws for neuronal avalanche and subsampled model avalanches

The above mentioned avalanche size distribution *f(s)* is only one of a set of scaling laws that characterize the dynamics of a system near criticality [8], [31]–[33]. Thus, a statement on the putative criticality of a system should be based on more than the scaling law for avalanche sizes. Therefore, we tested whether additional scaling laws held for neural avalanches and the subsampled SOC model. If both systems are near criticality, such additional scaling laws hold for the avalanche duration *d*, the inter avalanche intervals (*IAI*), and the shape function of an avalanche *F(t/d)*. *F(t/d)* describes the *scaled* number of events at time *t* within a single avalanche of duration *d*, and is expected to relate to the *total* number of events *S(t,d)* within an avalanche as follows:
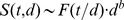
where *b* is the critical exponent. The critical exponents of all these scaling laws follow certain relationships (for more details about scaling laws and their interrelations see [32]–[34]).

Interestingly, the results for *f(d)*, *f(IAI)*, and *S(t,d)* were similar for the neuronal avalanches and the subsampled model avalanches (Figures S2, S3, S4). However, none of the distributions scaled as a power law. The deviations from power law scaling in the model were due to subsampling, since the fully sampled model follows the scaling laws [34], [35], and therefore subsampling may have caused the observed deviations from scaling laws in neuronal avalanches as well.

As a consequence the observed deviation from scaling laws for the neuronal avalanches does not allow us to reject the hypothesis that the human brain operates near criticality. In contrast - since the results for the neuronal avalanches and the subsampled model avalanches were qualitatively similar - we expect the neuronal avalanches to follow the scaling laws if sampled with even more electrode contacts.

### Neuronal avalanche size distributions differ between vigilance states

As mentioned before, the neuronal dynamics across vigilance states differed substantially (Figure S5). Indeed, vigilance states are classified into wake state, REM sleep, and non-REM (NREM) sleep stages by the characteristics of their neural mass signal, e.g. the sleep spindles, the slow waves, and sawtooth waves. For each vigilance state we therefore also calculated the avalanche distribution *f(s)* separately. We found that each of the avalanche distributions closely followed a power law with cutoff (Figure 4A), suggesting that neuronal dynamics of each of the vigilance states was close to the SOC state. Fitting of *f(s)* of each vigilance state to a power law with cutoff (eq. 1) resulted in τ = {1.52, 1.46, 1.24, 1.32} with α = {0.0064, 0.0169, 0.0573, 0.0415} from deep sleep to wakefulness.

**Figure 6:**
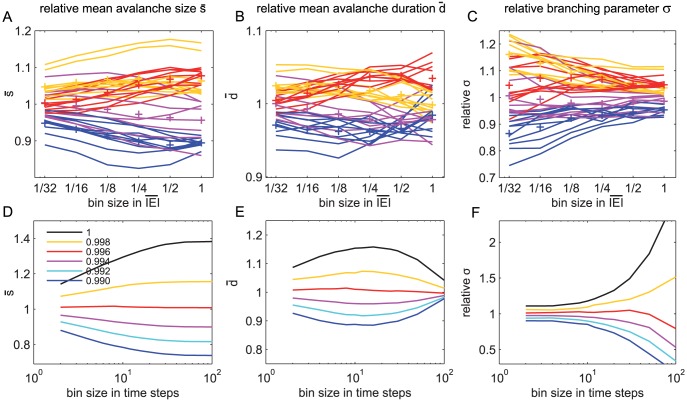
The avalanche measures all were larger with SWS in humans. **A–C** The avalanche measures (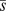, *đ*, and σ) were plotted over the bin size separately for each vigilance state (colours) and each night (traces). (+) indicate the mean measure across nights for each vigilance state. For SWS (s3/s4 and s2), neuronal avalanches were larger, longer and showed a larger branching parameter. The results here were shown for *r = ¼Hz*, however, the same results held for other rates (Supplementary Figure S6). **D–F** The same avalanche measures were plotted for the subsampled model. The model was varied from critical (black traces) to various degrees of subcriticality (*dE<1*). Subcritical models (*dE<1*) that were closer to the critical state (*dE = 1*) showed larger and longer avalanches, and larger branching parameter.

**Figure 7:**
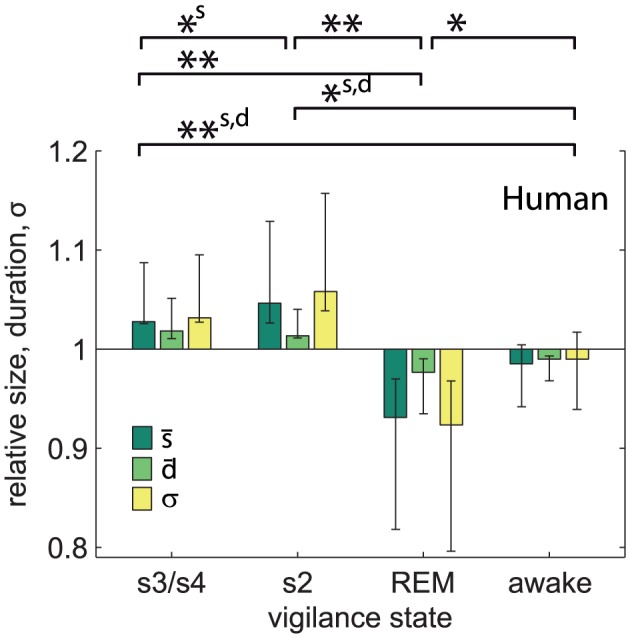
The avalanche measures differed between vigilance states. Each of the three measures (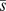, *đ*, and σ) was larger for SWS (s3/s4, s2) and smaller for REM sleep and wakefulness. For illustration purpose, we here combined the values of each measure across all patients, temporal bin sizes, and rates, although the statistical test distinguished between these parameters. Boxes indicate the median, and error bars indicate the 25^th^ and 75^th^ percentiles. The error bars are relatively wide, since the parameters (*bs* and *r*) influenced the avalanche measures. All three avalanche measures showed similar test results in the statistical test, therefore test results were indicated only once (* *p<0.05*, ** *p<0.001*, after sequential Bonferoni correction; ^s^ indicates “significant for 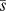 only”, and ^s,d^ “significant for 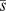 and *đ*, but not for σ”).

It is remarkable that the avalanche distribution *f(s)* approximated a power law for each of the vigilance states, given the differences in neuronal dynamics between these states. However, we also found that the avalanche distributions varied systematically across different vigilance states (Figure 4A,B). The distributions for REM decayed faster than those for SWS (s3/s4, and s2). This can be seen in *f(s)* for each single recording night (Figure 4B). To quantify these differences systematically, we calculated the *normalized* mean avalanche size 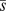 for each of the vigilance states at each parameter combination (*r, bs*). We used 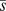 as a measure, since any change in 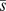 implies a change in *f(s)*. Figure 6A shows 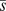 separately for each night and each state (colored) over the temporal scale (*bs*). 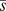 was always larger during SWS, and smaller during REM sleep. The same results held for the relative mean avalanche duration *đ* (Figure 6B), and the relative branching parameter σ (Figure 6C). All these results held across all event rates and temporal scales (Figure S6). To test these results for significance across all rates and temporal scales, we used a randomization statistic with cluster-based corrections for multiple comparisons [25]. We found that indeed all these three measures, 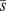, *đ*, and the relative σ, were significantly larger for SWS than for REM and the wake state (Figure 7). Thus, with deep sleep, the brain showed larger and longer neuronal avalanches with a larger branching parameter than during wakefulness and REM. Larger and longer avalanches correspond to stronger correlations between sites.

Independently of the framework of SOC, the differences in avalanche distributions with vigilance states reflected changes in the underlying correlation structure between brain areas. SWS thus showed stronger correlations between brain areas, in agreement with previous results [20], while REM to our surprise showed weaker correlations compared to wakefulness. The decrease of correlation strength with REM, compared to wakefulness and SWS, indicated more fragmented patterns of neuronal activity across cortical areas.

Here we showed that differences in neuronal activity between vigilance states were reflected in the avalanche distributions, although each of the distributions stayed close to a power law. In sum, the brain may operate close to SOC for any brain state from wakefulness to deep sleep, but still undergoes small but clear transitions with vigilance states. This naturally leads to the question about the underlying mechanisms that may change 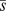, *đ*, and σ.

### Changes in neuronal avalanche distributions reflect deviations from criticality

We observed changes in the neuronal avalanche measures 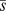, *đ* and σ with vigilance states, implying systematic changes in the avalanche distributions. Changes in the avalanche distributions can either be caused by *deviations* from power law scaling or by *changes in the critical exponent* τ (larger τ would lead to smaller 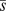). While the first indicates deviations from the criticality to sub- or supercritical regimes, the second indicates different critical states. Note that this also holds for high dimensional systems with “critical manifolds” instead of critical states: On these critical manifolds power law scaling holds while deviations from the critical manifolds are reflected in deviations from power law scaling [32], [33].

The first case, *deviations* from power law scaling, might occur in neural networks by changing the effective synaptic strength between units. For example, weakening the effective synaptic strength would impede avalanche propagation and tune a critical network to the subcritical regime. The second case, *changes in critical exponents*, can occur with changes in the topology of the model [8], [18]. For example, if one changes the topology of the 2D SOC model from next neighbor connectivity to random connectivity, while keeping the number and strength of connections fixed, τ increases from *1.1* to *1.4* [18].

To distinguish these two alternatives – deviations from power law scaling versus power law scaling with different exponents – we fitted *f(s)* to the following function:
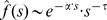
If α = *0*, 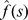 is a power law proper (with its best fitting τ), while α≠*0* indicates deviations from a power law. In this sense, α serves as a measure of criticality, since α quantifies the deviation from a power law distribution. Thus, a systematic change in α with vigilance states indicates systematic deviations from power law scaling.

We fitted *f(s)* to 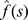 for each recording night, vigilance state, rate and bin size and found that the observed α was small (α = *0.043±0.065*), but it significantly depended on the vigilance state (cluster based randomization test on F-metric, *p<0.001* [25]). In detail, α was smallest for deep sleep (s3/s4 and s2), and largest for REM. These results are in line with the previous results on 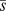, *đ* and σ (Figure 7). Differences in α were significant for all pairs of vigilance states except for s3/s4 versus s2 and REM versus wakefulness which both only showed a trend (trend: *p<0.08*; for all the other pair wise comparisons: *p<0.05*; post hoc cluster based randomization on T-metric [25], all *p* values were corrected for multiple comparison). Thus *f(s)* showed systematic deviations from power laws with vigilance states, indicating that brain dynamics indeed deviated from criticality.

### The variations in 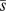, đ, and σ may arise from tiny changes in synaptic strength

For the neuronal avalanche size distribution in humans, we found small deviations from power law scaling with vigilance states, which were also reflected in the avalanche measures 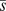, *đ*, and σ. These deviations from power law scaling likely reflect transitions away from criticality as pointed out above. Here we want to investigate how such changes in 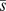, *đ*, and σ arise from a SOC model. In our model, transitions from sub- to supercriticality can be mediated by changes in effective synaptic strength *dE* [36], [37]. Such changes in *dE* during sleep were shown to have a direct impact on LFP dynamics in a large scale model [38]. In our model, we applied very tiny changes of *dE* around the critical state (*dE = 1*). Indeed, an increase in *dE* resulted in more large and longer avalanches with a larger branching parameter (Figure 4C,D). We quantified these effects systematically, using the relative measures 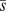, *đ*, and σ (Figure 6D–F). These measures all became smaller with smaller *dE*, independent of the bin size. Qualitatively, the changes of the relative measures in our model avalanches and in the neuronal avalanches from humans were similar (Figure 6). Thus the differences in neuronal avalanches between vigilance states may be mediated by tiny changes in the effective synaptic strength *dE*.

Note that the changes in *dE* in the model were very small, less than 1%, but nevertheless caused major changes in the avalanche measures, as expected near criticality. This in turn suggests for the human brain that the effective synaptic strength remains within a very narrow range from wakefulness to deep sleep.

## Discussion

For the neuronal avalanches from humans, we found on the one hand approximate power law scaling for each of the vigilance states, on the other hand we found significant differences between the *f(s)*, *f(d)* and σ with vigilances states. These differences reflected deviations from criticality rather than different critical states (see results section). This naturally leads to the question, how the vigilance states are mapped on critical, sub- and supercritical dynamics.

An ad hoc hypothesis may be that SWS was slightly supercritical, because it showed the largest avalanche measures; the wake state might then be closest to criticality, and REM, which showed the smallest avalanche measures, was in the subcritical regime. Supercritical states were observed for cortical slices and manifested as increased fraction of avalanches which span all recording sites (*s≈N*) [8], and in SOCM and supercritical branching processes they were characterized by an increased number of large avalanches with *s>N* (Figure 4C, red traces) [39]. However, for SWS we did not observe any of the characteristic features of the supercritical regime. We neither observed a peak in *f(s)* around *s≈N≈50* nor an increased number of large avalanches with *s>50* (Figure 4A). Therefore, we suggest that in the context of criticality, the human brain still operates very close to the critical state but in a subcritical regime. Supporting evidence for this interpretation is provided by the subcritical branching parameter (Figure 3D).

The above hypothesis is fully in line with results on α, a measure for the deviation from power law scaling: α was closest to zero for SWS, indicating that SWS was closest to the critical state. α was larger (and positive) for REM and wakefulness, indicating deviations from criticality to the subcritical regime. More precisely, α was largest for REM, intermediate for wakefulness and smallest for SWS, which is in agreement with the results for the avalanche measures discussed above. Based on α, the avalanche measures and the lack of evidence for supercriticality, we suggest that the human brain operates very close to the critical state but in a subcritical regime, where SWS is most close to criticality, wakefulness is slightly more subcritical, and REM sleep is even more subcritical.

One may argue that biological systems are not physical models and in real world biological systems small changes between subcritical and critical states, based on putative changes in *dE* of ∼0.1% might be negligible. However, these small changes in *dE* had major impact on the avalanche dynamics in the modified SOC models (manifested in 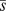, *đ*, and σ). Moreover, in our data, the differences with vigilance states were highly consistent across the ten recording nights (Figure 4B). Therefore, we do not attribute them to biological variability.

The differences in avalanche measures were not caused by a trivial change in the amplitude of the LFP with vigilance states. An increase in LFP amplitude is indeed observed from wakefulness to deep sleep, and it would directly result in larger avalanches if a constant threshold across all vigilance states had been applied. However, we adjusted the threshold for each vigilance state separately such that each electrode contributed with same event rate *r*. Putative changes in the LFP amplitude are therefore *not* the cause for changes in avalanche measures with vigilance states. Instead, the avalanche measures directly reflect the global correlation structure of *enhanced activity* between recording sites or brain areas.

Since the differences in avalanche measures were highly systematic and our results point to a slightly subcritical mode of operation, we may ask how to reconcile this with theoretical considerations that stress the computational optimality of the critical state [2], [4], [40]. In this respect it is important to note that some of these studies actually argue for a mode of operation that is close to critical and thereby in line with our findings. Moreover, one recent study demonstrated optimal task performance in the subcritical regime, although network evolution started out in a critical state [41]. One explanation for the differences between the theoretical models is that Lazar's model [41] incorporated learning and structured input, which was missing from the earlier works. As learning and structured input are relevant to the brain, this suggest that a slightly subcritical regime for neuronal dynamics is in fact optimal.

In addition, computational optimality may not have been the only evolutionary constraint, but stability might have been an additional goal. Stability is compromised in the supercritical state as the supercritical state was linked to epileptic behaviour [19], [42], [43]. It may well be that the brain in all its vigilance states maintains a safety margin to the supercritical state, because supercriticality allows for runaway activity, which is pathological, energy demanding and may induce erroneous learning [42].

The idea that the brain maintains a safety margin to the critical state was also brought forward by Pearlmutter and Houghton [44]. In addition they proposed that during wakefulness the brain approaches the critical state, while during sleep the safety margin is re-established again. This is in line with Tononi's proposal of synaptic downscaling during sleep [13]. We tested on our data whether we find any evidence for synaptic re- scaling in the avalanche measures over the course of the night or within a single sleep cycle, but did not find any systematic effect across patients (results not shown). This can have a multitude of reasons, but for now our avalanche analyses could not confirm the synaptic rescaling hypothesis.

Despite our claim that the brain operates in the subcritical regime, the main differences of our study to previous ones are minor regarding the main findings with respects to avalanche distributions. We found that among the available distributions a power law distribution with cutoff best described the empirical avalanche distribution - in line with previous findings *in vivo* [23], [45]–[47]. However, the availability of brain states with distinct dynamics and the observation of small but consistent differences in their avalanche measures forced us to conclude that the brain cannot always operate in the critical state. Furthermore, detailed analysis of the avalanche distributions, the branching parameter and a comparison to modeling results led us to the conclusion that the brain in fact operates in the subcritical regime.

Our study differs from two previous studies in rats and humans which evaluated different vigilance states but did not report differences in avalanche dynamics [48], [49]. The differences with vigilance states may have remained hidden in the variability of the recordings, since the number of recording sites, neurons, and their firing rates have impact on the avalanche measures, and differences only became obvious after proper normalization (Figure 6).

While the differences between vigilance states were highly stable across recording nights and patients, we can only speculate about their underlying physiological mechanisms. A potential mechanism, suggested by our SOCM, is a change in effective synaptic strength. This change may well be mediated by the global action of neuromodulators, since neuromodulators, such as acetylcholine (ACh), influence vigilance states [50]–[52] and supposedly modify the correlation structure between brain areas [53]. In fact, basal forebrain ACh release showed the same dependence on vigilance states in other studies [54] as the avalanche sizes reported here: REM sleep showed the highest ACh levels and the largest avalanches, wakefulness intermediate ones, and SWS the smallest. We hypothesize that the observed fragmentation of avalanches in REM sleep was mediated by increased levels of ACh, as proposed in a model by Avella-Gonzalez and colleagues [53]. Regarding the observed increase in avalanche sizes with SWS, this may be linked to up- and down-states, which are typically synchronized across brain areas [55]. However, the precise action and especially the interaction of various neuromodulators in sleep have not been sorted out. In fact, their precise role has not even been fully understood in small systems with less then 30 cells [56], and it would be premature to draw strong conclusion on the relationship between neuromodulators and neuronal avalanches.

Independent of the details of modulator actions, our results suggest that the effective synaptic strength *dE* stays tuned to a very narrow range of operation from wakefulness to deep sleep. Remember that in the model a change in *dE* of 0.2% resulted in changes in the avalanche measures of ∼5% - an effect of a size that was similar to what we observed in our human data. The question how the neural network maintains itself in this very narrow dynamical range remains open.

Independent of the context of SOC, the analysis of neuronal avalanches serves as a very useful measure to characterize the global correlation structure in massively parallel recordings (Figure 1). It captures the spatio-temporal dynamics beyond pairwise interactions and therefore may become increasingly important for the analysis of multisite recordings. Applying these avalanche measures, we could confirm that LFP activity across brain areas shows enhanced correlations during SWS [20], [57], [58]. In contrast, and to our surprise, REM showed a decrease in global correlation strength compared to wakefulness. The association of REM with decreased correlations is to the best of our knowledge new. A decrease in correlation strength during some phase of sleep, however, has been proposed by theoretical studies about learning [59], [60]. We propose that this decorrelation takes place during REM sleep. In sum, the analysis of neuronal avalanches confirmed correlated dynamics across brain areas in SWS and revealed a new phenomenon, namely the fragmented dynamics of REM sleep. Interestingly, wakefulness did not take an extreme value but its brain dynamics stayed just between the “fragmented” REM and the “correlated” SWS.

To conclude, our analyses of avalanche dynamics from human intracranial depth recordings indicated that the human brain operates close to criticality from wakefulness to deep sleep, as indicated by a power-law like distribution of avalanche sizes for each vigilance state. However, the sizes of neuronal avalanches changed with vigilance states: SWS showed larger and longer avalanches, wakefulness showed intermediate ones, and REM showed smaller and shorter ones. The larger avalanches of SWS confirm the correlated character of SWS dynamics across brain areas, while the smaller avalanches of REM revealed a fragmented organization of brain dynamics compared to wakefulness and SWS. Comparisons to a SOC model composed of integrate and fire units suggest that these differences may arise from tiny changes in effective synaptic strength, and that – in the context of criticality – the brain undergoes transitions within the subcritical regime close to but not including the critical state proper.

## Methods

### Experimental procedures

#### Data recording and preprocessing

We analyzed data from five subjects (3 females (aged 21, 23, and 27), two males; (aged 25 and 48)) with refractory partial epilepsy undergoing presurgical evaluation. The subjects were hospitalized between February 2005 and March 2007 in the epilepsy unit at the Pitié-Salpêtrière hospital in Paris. All patients gave their informed consent and procedures were approved by the local ethical committee (CCP). Each patient was continuously recorded during several days (duration range, 9–20 days; mean duration, 16 days) with intracranial and scalp electrodes (Nicolet acquisition system, CA, US). Depth electrodes were composed of 4 to 10 cylindrical contacts 2.3-mm long, 1-mm in diameter, 10-mm apart center-to-center, mounted on a 1 mm wide flexible plastic probe. Pre and post implantation MRI scans were evaluated to anatomically locate each contact along the electrode trajectory. The placement of electrodes within each patient was determined solely by clinical criteria. Signals were digitized at 400 Hz. For sleep data, two seizure-free nights with at least two complete sleep cycles were chosen from each of the subjects; in addition, for wakefulness data, between one and four seizure free recording hours were chosen preceding or following the night (for eight out of the ten nights). For each night, sleep stages were scored using the software Somnologica Studio (Embla Systems, Inc, CO, USA) and scores were visually confirmed by a time-frequency analysis. The four sleep stages were: REM, s1, s2, and s3/s4. (Sleep stages s3 and s4 were combined to s3/s4 to adapt to the AASM standards (REM, N1, N2, N3) [61], and s1 could not be used for the analysis because not all patients showed sufficiently long s1 sleep intervals). These three sleep stages together with the wake state made up the four vigilance states.

The five subjects were implanted with (44, 48, 50, 50, and 63) intracranial LFP recording sites. In total 7 recording sites were excluded from the analysis due to artifacts and thus we used (44, 48, 45, 50, and 61) recordings sites for data evaluation. All LFP were lowpass filtered at 40 Hz (4^th^ order butterworth, MATLAB) to reduce the impact of line noise.

#### Event definition for avalanches

Neuronal avalanches are spatiotemporal clusters of events which are separated by phases of quiescence. In the following, we define the events, the phases of quiescence between avalanches and several avalanche measures, following closely the procedures of Beggs and Plenz [18], [19]. For the event definition, we calculated the area under the positive deflection lobes between two zero crossings of the LFP (Figure 2, box) [18]. As LFP-voltages reflect current flows via Ohm's law, this time integral, the area under the voltage curve, is proportional to the total amount of displaced charges and hence describes the departure from equilibrium (charge neutrality) quantitatively – in contrast to simple voltage peaks. To obtain binary events from the LFP, we applied a threshold to the area values under the LFP deflection lobe. The threshold was selected such that each recording site in each interval of constant vigilance state had the same event rate *r*. Thereby each site at each vigilance state had the same “chance” to contribute to the avalanches. We chose to fix the event rate and not the threshold, because a fixed threshold is sensitive to changes in the LFP on one electrode, while we were interested in the propagation pattern of waves of *enhanced activity* – independent of the precise LFP shape that depends on vigilance states and also might depend on local tissue properties. With imposing a fixed event rate, we can distinguish whether the avalanches are rather fragmented or span the entire system. To demonstrate that our results did not depend on a specific choice for the event rate, we used a range of rates *r* = {1/10 Hz; 1/4 Hz; 1/2 Hz; 1 Hz}.

#### Avalanches and avalanche measures

Avalanches composed of the events defined above were extracted separately for each phase of constant vigilance state that lasted at least 150 s. More specifically, to extract avalanches, we applied temporal binning. The time bins were defined in units of “average inter event intervals” 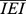. The 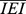 is a function of the event rate *r* defined above and the total number of recording sites *N*:
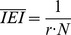
As an example, for *r* = *1Hz* and *N* = *50* electrodes, this resulted in 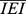 = 20 ms, while for *r* = *0.1Hz* and *N* = *50*, 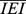 = 200 ms. We applied a large range of bin sizes *bs* = [1/32; 1/16; 1/8; 1/4; 1/2; 1; 2; 4] 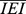.

Using this binning, an avalanche is defined as the cluster of events in subsequent non-empty time bins, and subsequent avalanches are separated by empty time bins. The avalanche size *s* is then the total number of binary events in an avalanche, and the avalanche duration *d* is the number of time bins it covers. The avalanche size distribution *f(s)* is the frequency distribution of avalanche sizes, as the avalanche duration distribution *f(d)* is the frequency distribution of durations. The corresponding probability distributions are *p(s)* and *p(d)*.

The above definitions of the bin size imposed practical limits on its range. The *bs* was limited on the lower end by the sampling rate resolution (*2.5 ms*) and on the upper end by the lack of pauses. In addition, for *bs>1* 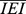, subsequent avalanches are “glued” together and one starts implicitly analyzing the temporal distribution of avalanches instead of the size distribution of single avalanches.

The avalanche size distribution *f(s)* was calculated for each night and for each sleep stage separately. To compare *f(s)* between sleep stages, we calculated the normalized mean avalanche size 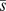 for each vigilance state,
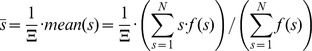
where *N* is the number of recording sites and Ξ is the normalization factor, namely the mean avalanche size over all vigilance states of one night:
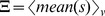


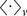
 denoted the mean over all vigilance states *v*. The normalization 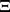 accounted for the difference in *N* across patients. 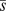 was calculated for each recording night, *bs* and r separately. The same was done to calculate the normalized mean avalanche duration *đ*.

In addition to the avalanche size and durations, we estimated the branching parameter σ. It describes whether activity expands (σ>*1*) or dies out (σ<*1*). For a single transition, σ′ was defined as the number of events in one time bin divided by the number of events in its preceding time bin. σ then is the average over all σ′ with non-zero preceding time bins. The normalized σ for each vigilance state *v* was calculated separately for each night, rate and *bs*. It was defined analog to 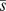 and *đ* as σ/<σ>*_v_*.

Further, the *IEI* was defined as the time interval between two subsequent events, taking into account all events across all channels. The distribution of *IEI* is denoted as *f(IEI)*. The relation between the *IEI* to the Inter Avalanche Interval *(IAI)* depends on the *bs* as follows: All *IEI* which are larger than *bs*·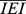 contribute to the *f(IAI)*.

Near criticality, the activity profile during a single avalanche is expected to show a characteristic shape *F(t/d)*, which simply scales with the avalanche duration *d* as follows: *S(t,d) = F(t/d) d^b^*, where *S(t,d)* is the number of events at time *t* in an avalanche of duration *d*, and *b* is a critical exponent (note that in criticality literature *b = 1*/σν*z-1* [31]–[33], but for simplicity we use *b* here). To test whether this relationship holds for the experimental data, we first obtained *S(t,d)* for each *d* (applying temporal bins). From these *S(t,d)* we obtained a collapse of all *S(t,d)* by rescaling the time axes to *t/d*, and rescaling the amplitude with a scaling factor *χ(d)* which was defined such that it minimized the absolute differences between the curves. If then the scaling factor *χ(d)* followed a power law relationship, *χ(d)*∼*d^b^*, this indicates that the system is close to criticality [8], [31].

We did the same analysis on the SOC model. For the fully sampled model it is straight forward to estimate avalanche size and duration. However, for the subsampled model a single “real” avalanche can appear, disappear and reappear on the subset of sites, leading to an observed time series of events, which showed pauses. On this time series, we applied temporal bins, just like for the experimental data, aligning the bins to random starting points to avoid any bias. We then analyzed these events the same way as for the experimental data: after applying temporal binning, we extracted the avalanche shapes *S(t,d)*, and estimated the scaling factor *χ(d)*. We analyzed this measure only for *d≥8* samples (which equals 20 ms at a rate of 400 Hz in the experiment), since shorter *d* have too few time points to derive the shape function *F(t/d)*.

In each patient, most electrode contacts were placed in the neocortex (NC), while a few were in the amygdala or hippocampus (AH). To test whether these groups of contacts contributed differently to the avalanches, we calculated for each contact *c* the probability *p_c_(s)* to participated in an avalanche of size *s* for *s = {1, N}*, where *N* is the total number of contacts. We then tested whether *p_c_(s)* for the NC contacts differed from *p_c_(s)* for the AH contacts across patients. We applied cluster based randomization on the T-metric as described by Maris and colleagues [25], (see below).

#### Statistical test

We tested whether the measures 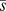, *đ*, and the relative σ varied with vigilance states. Since all these measures depended continuously on *bs* and *r*, we therefore analyzed the measures for all values for *bs* and *r*, and applied a cluster based correction for the arising multiple comparisons and randomization testing following Maris and colleagues [25]. Furthermore we separately analyzed both the normalized and non- normalized measures, and found the same qualitative results.

### Simulation procedures

#### Model description

The self-organized critical model (SOCM) we used here is the Bak-Tang-Wiesenfeld model [26]. It was run on a 3D grid of 25×25×25 = 15625 sites. Each site is connected with its six next neighbors. Each site (x,y,z) carries a certain level of energy, *E(x,y,z,t)* at time t, and if that level exceeds a threshold of six, it distributes *dE* = 1 to each of its six next neighbors in a process referred to as a toppling:
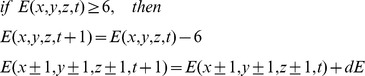
with *dE* = 1. (x±1,y±1,z±1) denote the 6 next neighbors of *(x,y,z)*. In the subsequent step after the toppling the *E* of the next neighbors may cross threshold and this neighbor will topple. This chain reaction triggers a spatiotemporal wave or avalanche which propagates through the 3D grid. Outside the grid *E* = 0 holds, i.e. open boundary conditions. If all sites on the grid have energy levels below threshold the avalanche terminates. A new avalanche can be triggered by adding one energy unit to a randomly selected site.

For this model, the size *s* of an avalanche is defined as the total number of topplings during a single avalanche. The frequency distribution *f(s)* of avalanche sizes shows a power law distribution [26]. We modified the SOCM as follows: We systematically changed the signal propagation efficacy *dE* (representing synaptic strength) between the model sites. When changing *dE*, the next neighbors received *dE<1* or *dE>1*. This shifted the dynamics of the model to subcritical and supercritical states, respectively.

#### Mimicking incomplete sampling of the brain in the model analysis

To increase model similarity to brain recordings, where we have only a limited number of recording sites, we in analogy sampled only a fraction of the sites of the SOC model, namely a centered, regular cube of 4×4×4 = 64 units with distance *2* between the sites. When subsampling, the avalanche size *s* was defined as the total number of events that occurred on the 4×4×4 selected sites during a single avalanche on the entire model.

#### Simulation of avalanche size distributions from 44 units with various types of correlations

The avalanche size distribution in a neural network depends on the correlation structure between the units. To demonstrate this effect, we ran *N = 44* units with varying correlation structures between their activity. All four examples are realizations of stochastic processes with an event rate of *r = ¼Hz* per unit. The stochastic processes were realized as follows: For the “independent” units, we ran *N = 44* independent Poisson processes with *r = ¼Hz*. For the “clustered” units, we defined two subsets with *N_1_ = 11* and *N_2_ = 33* units. Each of these subsets received independent stochastic “stimuli” with rate *r* at times *t*. The “stimuli” consisted of a transient rate increase described by a Gaussian probability distribution with *(25 ms)^2^* variance, centered on randomly drawn times *t*. For the “correlated” processes we realized a Poisson process with rate *r* for the first unit. Each subsequent unit had *99%* the same spike times as the previous and *1%* new spike times. The fourth process was not simulated but contained events of the recordings in humans, namely from the first patient (*N = 44* electrodes, *r = ¼Hz*). To estimate *f(s)*, we applied a bin size of *bs = 1* 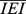 = *1/(r·44) = 0.91 s*.

#### Fitting of models to the avalanche distributions

We fitted all *f(s)* from the SOCM and the neuronal recordings to a power law proper, 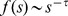, or a power law with cutoff
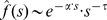
using maximum likelihood estimation (MLE) for the parameters τ and α based on the methods proposed by Clauset et al. [24], and modified for functions with cutoff by Klaus et al. [23]. As proposed by Clauset et al., we also tested alternative models to the power law proper, namely exponential, and Poisson, and as alternative models for the power law with cutoff, we used a log- normal distribution and a stretched exponential. To estimate, which of these models provided the highest model evidence, we calculated the maximum likelihood ratio *R* with respect to the likelihood of the power law proper [24].

#### Virtual LFP avalanches from the spiking SOC model

We sampled virtual LFP signals from the spiking SOC model (50×50×50 sites). We sampled with 4×4×4 virtual electrodes with distance 15 between the electrode tips. The virtual electrodes sampled from all sites of the model, weighted with a Gaussian kernel with variance 5, centered on the electrode tip (figure 5A). The analog signal on the virtual electrodes was processed similar to the LFP: We calculated the area under the deflection lobes between zeros and applied a range of thresholds [10^−4^], [10^−3^], [10^−2^], [10^−1^], [1], [10]. The resulting binary events formed avalanches which were extracted as described above.

## Supporting Information

Figure S1This figure relates to figure 3 and demonstrates the quality of power law fits to *f(s)*. The black trace depicts as one example the neuronal avalanche distributions *f(s)* for human data, with *r = ¼Hz* at *bs = 1* 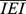, and the colored traces show the resulting best fits to *f(s)* on the interval *s =* [*1*], [50], for various functions as indicated in the legend. Most functions resulted in a close fit, only the Poisson and exponential showed strong deviations. The power law with cutoff (pink), however, provided the best fit (maximum likelihood ratio [Clauset et al., 2009], see also supplementary table S1).(EPS)

Figure S2 **A.** The distribution of the inter avalanche interval (*IAI*) changed with the underlying event rate. With higher rate, the *IAI* became smaller, since more events were distributed within the same recording time. *f(IAI)* is in absolute counts for both the combined data (colored), and for each individual recording night (grey). **B.** The *IAI* distribution for the subsampled SOC model was similar to the one from the human data.
(EPS)

Figure S3The avalanche duration distribution *f(d)* did not show a power law neither for the human data nor for the subsampled SOC model. **A.** *f(d)* from the human patients was similar for each event rate *r* (colored lines; *bs* = 1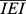). *f(d)* was in absolute counts, therefore, the offset between the *f(d)* reflected the higher number of events with higher *r*. The gray lines depict *f(d)* for each of the 10 recording nights separately with some offset to show that the variability between patients was very small. **B.** The avalanche duration *f(d)* changed with the bin size from *bs* = 1/32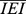 to *bs* = *4* 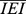
(at *r = ¼Hz*). However, for all *bs<1* 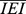, the maximal observed duration was ∼22, while larger *bs* also showed longer avalanche durations. The same was observed for the avalanche size distributions (figure 3). **C.** *f(d)*, sampled from 4×4×4 = 64 sites of the SOC model, did not show a power law, however, the distribution resembled the one for the experimental data, especially for small *bs*. For larger *bs*, the avalanches became shorter. This is due to the separation of time scales which is implemented in SOC models but not prominent in neuronal data.(EPS)

Figure S4Renormalization analysis on neuronal avalanches and model avalanches. **A.** The number of events *S(t,d)* within a single neuronal avalanche changed with time *t* (relative to the start of an avalanche) and depended on the avalanche duration *d*. Here we plotted *S(t,d)* for each vigilance states for three different durations *d* (40 ms, 80 ms and 120 ms; at *bs = ¼* 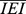 *≈20 ms* and event rate *r = ¼Hz*). **B.** The estimated shape functions *F(t/d)* were collapsed optimally using the same data as in A (shown for wakefulness). **C.** From the optimal collapse (B), the scaling factor *χ(d)* was estimated. From renormalization theory, *χ(d)* is expected to follow *χ(d)*∼*d^b^*, which is a straight line in the double logarithmic plot. However, this scaling broke down for larger *d*. This indicates that with larger *d* the amplitude of *S(t,d)* increased less than expected from the scaling relationship. Error bars in all plots indicate 25% and 75% percentiles from boot strapping. **D–F** The same as in A–C, however, for the subsampled model, using the usual subset (4×4×4 sites with distance 2). The scaling in the subsampled model showed similar results like the experiment: The collapse of the *S(t,d)* to the estimated shape function *F(t,d)* worked well (E), however, the estimated scaling factor *χ(d)* dropped for large *d* (F). This reflected subsampling effects in the model. **G–I.** The same as in A–C but for the fully sampled model. Here *χ(d)* followed a straight line for avalanches up to *d∼100*. This indicated that the fully sampled model obeyed scaling relationships, while the deviations for *d>100* may be due to finite size effects.(EPS)

Figure S5 This figure relates to the main figure 4 and shows how neuronal activity differs between vigilance states. **A.** The hypnogram indicates the sleep stage at each time during a complete night. The sleep stages occur normally in a characteristic order, called sleep cycle, which goes from s1 to s2, s3/s4, back to s2 and then REM. **B.** The corresponding time-frequency plot for one scalp EEG electrode shows the different frequency activities related to each vigilance state. **C.** The wake state shows pronounced α rhythm (8–13 Hz). **D.** The light sleep stages (s1,s2) show sleep spindles and K-complexes. **E.** The deep sleep (s3/s4) shows synchronized slow waves (<4 Hz). **F.** The REM stage is characterized rapid eye movements (not shown) and by low-voltage activity.
(EPS)

Figure S6 This figure relates to the main figure 6. It shows the same measures, namely the relative mean size and duration, and the relative branching parameter, however, it shows the measures for all event rates (top to bottom: 0.1Hz, 0.25Hz 0.5Hz and 1Hz). All the three measures were here plotted for each recording night, bin size (x-axes), and rate separately. (+) indicate the mean across all recording nights. For better visualization, we plotted the *relative* measures, which were defined as relative change in a measure across vigilance states for each patient at a certain bin size and rate. For most parameters, SWS showed larger avalanche measures than REM sleep. Wakefulness tended to show intermediate values. (Note that for large event rates (bottom two rows), values at small bin sizes are not defined, because the bin width was smaller than the sampling rate interval.)
(EPS)

Table S1 This table relates to the main figure 3 and to the supplementary figure S1, and provides information on the quality of fits to *f(s)*. To test, whether a power law or an alternative function provided the best fit to the avalanche distributions *f(s)*, we fitted various functions (power law, Poisson, exponential, log- normal, stretched exponential, and power law with cutoff) to *f(s)* from the human data and the self- organized critical model (SOCM). The fit quality relative to the power law proper is indicated by *R*, the log likelihood ratio. A negative *R* indicates a better fit than the power law proper. For each *f(s)*, *R* was smallest for the power law with cutoff (last column, marked bold), indicating that the power law with cutoff provided the best fit. However, not even the power law with cutoff provided a sufficiently good fit to *f(s)* (Kolmogorov-Smirnov goodness of fit test (KS test), indicated in the last column: ‘none’ indicates *p<0.01* for the KS test). This held for all *f(s)*, being it from the human data or the SOC model. That the SOC model did not pass the test was very much to our surprise since the SOC model is “known” to show a power law for *f(s)*. In principle, finding a function that would pass the KS test could have been achieved by using more functions with more free parameters. However, this might not teach us more about neuronal avalanches. To keep it simple, and since the differences between the bivariate fits were small (figure and table), we opted to use both, a power law proper and a power law with cutoff as model functions for the characterization of the avalanche distributions – keeping in mind that neither the neuronal avalanches nor the SOC model avalanches followed a power law in the strict sense.
(EPS)

## References

[1] Tononi G, Koch C (2008) The neural correlates of consciousness: an update. Ann N Y Acad Sci 1124: 239–261 doi:10.1196/annals.1440.004 18400934 10.1196/annals.1440.004

[2] Bertschinger N, Natschläger T (2004) Real-Time Computation at the Edge of Chaos in Recurrent Neural Networks. Neural Computation 16: 1413–1436 doi:10.1162/089976604323057443 15165396 10.1162/089976604323057443

[3] Shew WL, Yang H, Yu S, Roy R, Plenz D (2011) Information Capacity and Transmission Are Maximized in Balanced Cortical Networks with Neuronal Avalanches. J Neurosci 31: 55–63 doi:10.1523/JNEUROSCI.4637-10.2011 21209189 10.1523/JNEUROSCI.4637-10.2011 PMC3082868

[4] Haldeman C, Beggs JM (2005) Critical Branching Captures Activity in Living Neural Networks and Maximizes the Number of Metastable States. Phys Rev Lett 94: 058101 doi:10.1103/PhysRevLett.94.058101 15783702 10.1103/PhysRevLett.94.058101

[5] Levina A, Herrmann JM, Geisel T (2007) Dynamical synapses causing self-organized criticality in neural networks. Nat Phys 3: 857–860 doi:10.1038/nphys758

[6] Millman D, Mihalas S, Kirkwood A, Niebur E (2010) Self-organized criticality occurs in non-conservative neuronal networks during Up states. Nature Physics 6: 801–805 doi:10.1038/nphys1757 21804861 10.1038/nphys1757 PMC3145974

[7] Rubinov M, Sporns O, Thivierge J-P, Breakspear M (2011) Neurobiologically Realistic Determinants of Self-Organized Criticality in Networks of Spiking Neurons. PLoS Comput Biol 7: e1002038 doi:10.1371/journal.pcbi.1002038 21673863 10.1371/journal.pcbi.1002038 PMC3107249

[8] Friedman N, Ito S, Brinkman BAW, Shimono M, DeVille REL, et al (2012) Universal Critical Dynamics in High Resolution Neuronal Avalanche Data. Phys Rev Lett 108: 208102 doi:10.1103/PhysRevLett.108.208102 23003192 10.1103/PhysRevLett.108.208102

[9] Pajevic S, Plenz D (2009) Efficient Network Reconstruction from Dynamical Cascades Identifies Small-World Topology of Neuronal Avalanches. PLoS Comput Biol 5: e1000271 doi:10.1371/journal.pcbi.1000271 19180180 10.1371/journal.pcbi.1000271 PMC2615076

[10] Adam K, Oswald I (1977) Sleep is for tissue restoration. J R Coll Physicians Lond 11: 376–388. 328867 PMC5368747

[11] McGinty D, Szymusiak R (1990) Keeping cool: a hypothesis about the mechanisms and functions of slow-wave sleep. Trends in neurosciences 13: 480–487. 1703678 10.1016/0166-2236(90)90081-k

[12] Maquet P (2001) The role of sleep in learning and memory. Science 294: 1048–1052. 11691982 10.1126/science.1062856

[13] Tononi G, Cirelli C (2006) Sleep function and synaptic homeostasis. Sleep Med Rev 10: 49–62 doi:10.1016/j.smrv.2005.05.002 16376591 10.1016/j.smrv.2005.05.002

[14] Scharf MT, Naidoo N, Zimmerman JE, Pack AI (2008) The energy hypothesis of sleep revisited. Progress in Neurobiology 86: 264–280 doi:10.1016/j.pneurobio.2008.08.003 18809461 10.1016/j.pneurobio.2008.08.003 PMC2948963

[15] Harris TE (1964) The Theory of Branching Processes. Springer - Verlag OHG. 256 p.

[16] Mazzoni A, Broccard FD, Garcia-Perez E, Bonifazi P, Ruaro ME, et al (2007) On the Dynamics of the Spontaneous Activity in Neuronal Networks. PLoS ONE 2: e439 doi:10.1371/journal.pone.0000439 17502919 10.1371/journal.pone.0000439 PMC1857824

[17] Benayoun M, Cowan JD, Van Drongelen W, Wallace E (2010) Avalanches in a Stochastic Model of Spiking Neurons. PLoS Comput Biol 6: e1000846 doi:10.1371/journal.pcbi.1000846 20628615 10.1371/journal.pcbi.1000846 PMC2900286

[18] Priesemann V, Munk MHJ, Wibral M (2009) Subsampling effects in neuronal avalanche distributions recorded in vivo. BMC Neurosci 10: 40 doi:10.1186/1471-2202-10-40 19400967 10.1186/1471-2202-10-40 PMC2697147

[19] Beggs JM, Plenz D (2003) Neuronal avalanches in neocortical circuits. J Neurosci 23: 11167–11177. 14657176 10.1523/JNEUROSCI.23-35-11167.2003 PMC6741045

[20] Volgushev M, Chauvette S, Mukovski M, Timofeev I (2006) Precise Long-Range Synchronization of Activity and Silence in Neocortical Neurons during Slow-Wave Sleep. J Neurosci 26: 5665–5672 doi:10.1523/JNEUROSCI.0279-06.2006 16723523 10.1523/JNEUROSCI.0279-06.2006 PMC6675259

[21] Buzsaki G, Traub RD (2008) Physiological Basis of the Electroencephalogram and Local field Potentials. In: Engel J, Pedley TA, editors. Epilepsy: a comprehensive textbook. Philadelphia: Lippincott-Raven Press. pp. 797–808.

[22] Buzsáki G, Anastassiou CA, Koch C (2012) The origin of extracellular fields and currents - EEG, ECoG, LFP and spikes. Nat Rev Neurosci 13: 407–420 doi:10.1038/nrn3241 22595786 10.1038/nrn3241 PMC4907333

[23] Klaus A, Yu S, Plenz D (2011) Statistical Analyses Support Power Law Distributions Found in Neuronal Avalanches. PLoS ONE 6: e19779 doi:10.1371/journal.pone.0019779 21720544 10.1371/journal.pone.0019779 PMC3102672

[24] Clauset A, Shalizi CR, Newman MEJ (2009) Power-Law Distributions in Empirical Data. SIAM Review 51: 661 doi:10.1137/070710111

[25] Maris E, Schoffelen J-M, Fries P (2007) Nonparametric statistical testing of coherence differences. J Neurosci Methods 163: 161–175 doi:10.1016/j.jneumeth.2007.02.011 17395267 10.1016/j.jneumeth.2007.02.011

[26] Bak Tang, Wiesenfeld (1987) Self-organized criticality: An explanation of the 1/f noise. Phys Rev Lett 59: 381–384. 10035754 10.1103/PhysRevLett.59.381

[27] Perin R, Berger TK, Markram H (2011) A synaptic organizing principle for cortical neuronal groups. Proceedings of the National Academy of Sciences 108: 5419–5424 doi:10.1073/pnas.1016051108 10.1073/pnas.1016051108 PMC3069183 21383177

[28] Yu S, Huang D, Singer W, Nikolic D (2008) A small world of neuronal synchrony. Cereb Cortex 18: 2891–2901 doi:10.1093/cercor/bhn047 18400792 10.1093/cercor/bhn047 PMC2583154

[29] Drossel B, Schwabl F (1992) Self-organized critical forest-fire model. Phys Rev Lett 69: 1629–1632 doi:10.1103/PhysRevLett.69.1629 10046273 10.1103/PhysRevLett.69.1629

[30] Bak P, Sneppen K (1993) Punctuated equilibrium and criticality in a simple model of evolution. Phys Rev Lett 71: 4083–4086 doi:10.1103/PhysRevLett.71.4083 10055149 10.1103/PhysRevLett.71.4083

[31] Sethna JP, Dahmen KA, Myers CR (2001) Crackling noise. Nature 410: 242–250 doi:10.1038/35065675 11258379 10.1038/35065675

[32] Stanley HE (1971) Introduction to phase transitions and critical phenomena. Oxford: Oxford University Press. 348 p.

[33] Henkel M, Hinrichsen H, Lübeck S (2009) Non-Equilibrium Phase Transitions: Volume 1: Absorbing Phase Transitions. Dordrecht: Springer. 396 p.

[34] Dhar D (2006) Theoretical studies of self-organized criticality. Physica A: Statistical Mechanics and its Applications 369: 29–70 doi:10.1016/j.physa.2006.04.004

[35] Jensen HJ (1998) Self-Organized Criticality: Emergent Complex Behavior in Physical and Biological Systems. 3rd ed. Cambridge: Cambridge University Press. 168 p.

[36] Bröker H-M, Grassberger P (1997) Random neighbor theory of the Olami-Feder-Christensen earthquake model. Phys Rev E 56: 3944–3952 doi:10.1103/PhysRevE.56.3944

[37] De Carvalho JX, Prado CPC (2000) Self-Organized Criticality in the Olami-Feder-Christensen Model. Phys Rev Lett 84: 4006–4009 doi:10.1103/PhysRevLett.84.4006 11019261 10.1103/PhysRevLett.84.4006

[38] Esser SK, Hill SL, Tononi G (2007) Sleep Homeostasis and Cortical Synchronization: I. Modeling the Effects of Synaptic Strength on Sleep Slow Waves. Sleep 30: 1617–1630. 18246972 10.1093/sleep/30.12.1617 PMC2276134

[39] Taylor TJ, Hartley C, Simon PL, Kiss IZ, Berthouze L (2012) Identification of criticality in neuronal avalanches: I. A theoretical investigation of the non-driven case. arXiv:12108295. Available:http://arxiv.org/abs/1210.8295. Accessed 11 December 2012. 10.1186/2190-8567-3-5 PMC3679959 23618010

[40] Maass W, Natschläger T, Markram H (2004) Fading memory and kernel properties of generic cortical microcircuit models. Journal of Physiology-Paris 98: 315–330 doi:10.1016/j.jphysparis.2005.09.020 10.1016/j.jphysparis.2005.09.020 16310350

[41] Lazar A, Pipa G, Triesch J (2009) SORN: A Self-Organizing Recurrent Neural Network. Front Comput Neurosci 3: 2–23 doi:10.3389/neuro.10.023.2009 19893759 10.3389/neuro.10.023.2009 PMC2773171

[42] Hsu D, Chen W, Hsu M, Beggs JM (2008) An open hypothesis: Is epilepsy learned, and can it be unlearned? Epilepsy & Behavior 13: 511–522 doi:10.1016/j.yebeh.2008.05.007 18573694 10.1016/j.yebeh.2008.05.007 PMC2611958

[43] Hobbs JP, Smith JL, Beggs JM (2010) Aberrant Neuronal Avalanches in Cortical Tissue Removed From Juvenile Epilepsy Patients. Journal of Clinical Neurophysiology 27: 380–386 doi:10.1097/WNP.0b013e3181fdf8d3 21076327 10.1097/WNP.0b013e3181fdf8d3

[44] Pearlmutter BA, Houghton CJ (2009) A New Hypothesis for Sleep: Tuning for Criticality. Neural Computation 21: 1622–1641 doi:10.1162/neco.2009.05-08-787 19191602 10.1162/neco.2009.05-08-787

[45] Petermann T, Thiagarajan TC, Lebedev MA, Nicolelis MAL, Chialvo DR, et al (2009) Spontaneous cortical activity in awake monkeys composed of neuronal avalanches. PNAS 106: 15921–15926 doi:10.1073/pnas.0904089106 19717463 10.1073/pnas.0904089106 PMC2732708

[46] Hahn G, Petermann T, Havenith MN, Yu S, Singer W, et al (2010) Neuronal avalanches in spontaneous activity in vivo. J Neurophysiol 104: 3312–3322 doi:10.1152/jn.00953.2009 20631221 10.1152/jn.00953.2009 PMC3007625

[47] Tagliazucchi E, Balenzuela P, Fraiman D, Chialvo DR (2012) Criticality in Large-Scale Brain fMRI Dynamics Unveiled by a Novel Point Process Analysis. Front Physiol 3 Available:http://www.ncbi.nlm.nih.gov/pmc/articles/PMC3274757/. Accessed 10 July 2012. 10.3389/fphys.2012.00015 PMC3274757 22347863

[48] Ribeiro TL, Copelli M, Caixeta F, Belchior H, Chialvo DR, et al (2010) Spike Avalanches Exhibit Universal Dynamics across the Sleep-Wake Cycle. PLoS ONE 5: e14129 doi:10.1371/journal.pone.0014129 21152422 10.1371/journal.pone.0014129 PMC2994706

[49] Dehghani N, Hatsopoulos NG, Haga ZD, Parker RA, Greger B, et al. (2012) Avalanche analysis from multi-electrode ensemble recordings in cat, monkey and human cerebral cortex during wakefulness and sleep. arXiv:12030738. Available:http://arxiv.org/abs/1203.0738. Accessed 5 July 2012. 10.3389/fphys.2012.00302 PMC3429073 22934053

[50] Tsodyks MV, Markram H (1997) The neural code between neocortical pyramidal neurons depends on neurotransmitter release probability. PNAS 94: 719–723. 9012851 10.1073/pnas.94.2.719 PMC19580

[51] Davis KL, Charney D, Coyle JT, Nemeroff C (2002) Neuropsychopharmacology: The Fifth Generation of Progress: an Official Publication of the American College of Neuropsychopharmacology. Philadelphia: Lippincott Williams & Wilkins. 2009 p.

[52] Mark S, Tsodyks M (2012) Population spikes in cortical networks during different functional states. Front Comput Neurosci 6: 43 Available:http://www.ncbi.nlm.nih.gov/pmc/articles/PMC3396090/. Accessed 9 August 2012. 22811663 10.3389/fncom.2012.00043 PMC3396090

[53] Avella Gonzalez OJ, Van Aerde KI, Van Elburg RAJ, Poil S-S, Mansvelder HD, et al (2012) External Drive to Inhibitory Cells Induces Alternating Episodes of High- and Low-Amplitude Oscillations. PLoS Comput Biol 8: e1002666 doi:10.1371/journal.pcbi.1002666 22956901 10.1371/journal.pcbi.1002666 PMC3431298

[54] Vazquez J, Baghdoyan HA (2001) Basal forebrain acetylcholine release during REM sleep is significantly greater than during waking. Am J Physiol Regul Integr Comp Physiol 280: R598–R601. 11208592 10.1152/ajpregu.2001.280.2.R598

[55] SIROTA A, BUZSÁKI G (2005) Interaction between neocortical and hippocampal networks via slow oscillations. Thalamus Relat Syst 3: 245–259 doi:10.1017/S1472928807000258 18185848 10.1017/S1472928807000258 PMC2180396

[56] Marder E, Thirumalai V (2002) Cellular, synaptic and network effects of neuromodulation. Neural Networks 15: 479–493 doi:10.1016/S0893-6080(02)00043-6 12371506 10.1016/s0893-6080(02)00043-6

[57] Quyen MLV, Staba R, Bragin A, Dickson C, Valderrama M, et al (2010) Large-Scale Microelectrode Recordings of High-Frequency Gamma Oscillations in Human Cortex during Sleep. J Neurosci 30: 7770–7782 doi:10.1523/JNEUROSCI.5049-09.2010 20534826 10.1523/JNEUROSCI.5049-09.2010 PMC3842470

[58] Botella-Soler V, Valderrama M, Crépon B, Navarro V, Le Van Quyen M (2012) Large-Scale Cortical Dynamics of Sleep Slow Waves. PLoS ONE 7: e30757 doi:10.1371/journal.pone.0030757 22363484 10.1371/journal.pone.0030757 PMC3281874

[59] Hinton GE, Dayan P, Frey BJ, Neal RM (1995) The “wake-sleep” algorithm for unsupervised neural networks. Science 268: 1158–1161 doi:10.1126/science.7761831 7761831 10.1126/science.7761831

[60] Weber C, Triesch J (2008) A Sparse Generative Model of V1 Simple Cells with Intrinsic Plasticity. Neural Computation 20: 1261–1284 doi:10.1162/neco.2007.02-07-472 18194109 10.1162/neco.2007.02-07-472

[61] Iber C, Ancoli-Israel S, Chesson AL, Quan SF (2007) The New Sleep Scoring Manual–The Evidence Behind The Rules. Journal of Clinical Sleep Medicine 3: 107.

